# Localization Patterns of RAB3C Are Associated with Murine and Human Sperm Formation

**DOI:** 10.3390/medicina58101408

**Published:** 2022-10-07

**Authors:** Yieh-Loong Tsai, Tsung-Hsuan Lai, Hsuan-Che Liu, Ya-Yun Wang, Yu-Hua Lin, Chih-Chun Ke, Ming-Ting Chung, Chying-Chyuan Chan, Ying-Hung Lin

**Affiliations:** 1 Department of Obstetrics and Gynecology, Shin Kong Wu Ho-Su Memorial Hospital, Taipei 111, Taiwan; 2 College of Medicine, Fu Jen Catholic University, New Taipei City 242, Taiwan; 3 Department of Obstetrics and Gynecology, Cathay General Hospital, Taipei 106, Taiwan; 4 Graduate Institute of Biomedical and Pharmaceutical Science, Fu Jen Catholic University, New Taipei City 242, Taiwan; 5 Department of Chemistry, Fu Jen Catholic University, New Taipei City 242, Taiwan; 6 Division of Urology, Department of Surgery, Cardinal Tien Hospital, New Taipei City 231, Taiwan; 7 Ph.D Program in Nutrition and Food Science, Fu Jen Catholic University, New Taipei City 242, Taiwan; 8 Department of Urology, En Chu Kong Hospital, New Taipei City 237, Taiwan; 9 Center for Reproductive Medicine, Chi Mei Medical Center, Tainan 710, Taiwan; 10 Department of Obstetrics and Gynecology, Taipei City Hospital, Renai Branch, Taipei 106, Taiwan

**Keywords:** teratozoospermia, SEPT14, RAB3C, sperm head defect

## Abstract

*Background and Objectives*: Septins (SEPTs) are highly conserved GTP-binding proteins and the fourth component of the cytoskeleton. Polymerization of SEPTs contributes to several critical cellular processes such as cytokinesis, cytoskeletal remodeling, and vesicle transportation. In our previous study, we found that SEPT14 mutations resulted in teratozoospermia with >87% sperm morphological defects. SEPT14 interactors were also identified through proteomic assays, and one of the peptides was mapped to RAB3B and RAB3C. Most studies on the RAB3 family have focused on RAB3A, which regulates the exocytosis of neurotransmitters and acrosome reactions. However, the general expression and patterns of the RAB3 family members during human spermatogenesis, and the association between RAB3 and teratozoospermia owing to a SEPT14 mutation, are largely unknown. *Materials and Methods*: Human sperm and murine male germ cells were collected in this study and immunofluorescence analysis was applied on the collected sperm. *Results*: In this study, we observed that the RAB3C transcripts were more abundant than those of RAB3A, 3B, and 3D in human testicular tissues. During human spermatogenesis, the RAB3C protein is mainly enriched in elongated spermatids, and RAB3B is undetectable. In mature human spermatozoa, RAB3C is concentrated in the postacrosomal region, neck, and midpiece. The RAB3C signals were delocalized within human spermatozoa harboring the *SEPT14* mutation, and the decreased signals were accompanied by a defective head and tail, compared with the healthy controls. To determine whether RAB3C is involved in the morphological formation of the head and tail of the sperm, we separated murine testicular tissue and isolated elongated spermatids for further study. We found that RAB3C is particularly expressed in the manchette structure, which assists sperm head shaping at the spermatid head, and is also localized at the sperm tail. *Conclusions*: Based on these results, we suggest that the localization of RAB3C proteins in murine and human sperm is associated with *SEPT14* mutation-induced morphological defects in sperm.

## 1. Introduction

### 1.1. Male Infertility and Spermatogenesis

Infertility has been recognized as a global public health concern by the World Health Organization, and it affects approximately 9% of couples worldwide [1,2]. Male infertility is responsible for 20–50% of infertility cases [3]. The pathological causes of male infertility include anatomic imperfections, gametogenesis abnormalities, endocrine dysregulation, aberrant immunological responses, ejaculatory defects, environmental toxicity, and genetic alterations [4,5]. Over the past two decades, assisted reproductive technology (ART), including in vitro fertilization (IVF) and intracytoplasmic sperm injection (ICSI), has been effective, and this technology has been adapted for infertile couples [6,7]. However, teratozoospermia is a currently unsolvable issue, even with ART. It is frequently accompanied by sperm DNA damage and negatively affects pregnancy outcomes and embryo progression, including recurrent spontaneous abortion, pregnancy failure, and lower live birth rates [8,9,10,11,12].

### 1.2. Septins

Septins are evolutionarily conserved GTP-binding proteins and are the fourth component of the cytoskeleton [13,14]. The polymerized SEPTs form hetero-oligomeric filaments or rings through incorporating with other cytoskeletal components to regulate cellular processes, such as cytokinesis, membrane dynamics, and vesicle traffic [15,16,17,18,19]. Dysregulation of SEPTs is associated with the molecular pathology of several types of cancer, neurological illnesses, and male infertility [19,20]. The reproductive and pathological roles of SEPT4, SEPT12, and SEPT14 are important in the late stage of spermatogenesis. For example, Sept4-null mice are infertile because of defective tails and immotile sperm [21,22]. Additionally, the localization and expression patterns of SEPT4 are diagnostic markers of human asthenozoospermia [23,24]. Loss of SEPT12 causes impaired sperm head and tail structures and is accompanied by DNA damage in human beings and mice [25,26,27]. In addition, the embryos in mice fertilized with Sept12-deficient sperm through intracytoplasmic sperm injection (ICSI) underwent developmental arrest at the early morula stage [28]. In our previous studies, we also observed that two SEPT14 mutations cause teratozoospermia, with defects mainly in the sperm head [29]. Furthermore, we identified SEPT14 interactors through co-immunoprecipitation and nano-liquid chromatography-mass spectrometry/mass spectrometry [30]. One such peptide belongs to RAB3B and RAB3C; the amino acid sequences of RAB3B and RAB3C are highly similar. 

### 1.3. Biological Roles of the RAB3 Family

RABs belong to the largest family of small Ras-like GTPases and currently have more than 60 members [31,32]. RABs control intracellular membrane trafficking, cell division, cell signaling, cell survival, and migration, and they relate to human diseases [33,34]. Within the RAB3 family, RAB3A was first found to be abundant in synaptic vesicles in the brain [35]. Geoppert et al. observed in Rab3a-null mice that Rab3A appears inessential for synaptic vesicle exocytosis; however, it plays a role in the enrollment of synaptic vesicles for exocytosis in hippocampal CA1 pyramidal cells [36,37]. Further, Rim has been identified as RAB3A effectors in regulating synaptic vesicle fusion using a yeast two-hybrid system [38]. Furthermore, RAB3B and RAB3C are also expressed in the brain and endocrine tissues [39]. RAB3A, RAB3B, and RAB3C colocalized to synaptic and secretory vesicles, which may involve a similar process with potential redundancy. However, RAB3D was expressed at high levels only in the endocrine glands, such as the parotid gland and pancreas. Riedel et al. (2002) observed that the size of the secretory granules in the exocrine pancreas and parotid gland of Rab3d-knockout mice was significantly enhanced to a doubled amount [40]. In reproductive biology, RAB3A is involved in the acrosome reaction, a membrane-fusion event that facilitates sperm penetration into the zona pellucida [41,42]. 

However, no other relevant studies have reported the roles of the RAB3 family in murine and human spermatogenesis, and the association between RAB3C and teratozoospermia caused by a SEPT14 mutation. In this study, we focused on the expression patterns of RAB3 family proteins and the association between RAB3 proteins and sperm morphology.

## 2. Materials and Methods

### 2.1. Sequencing Alignment and Data Mining of RAB3 Expression in Human Testis

Alignment of the amino acid sequences of human RAB3A (NP_002857.1), RAB3B (NP_002858.2), RAB3C (NP_612462.1), and RAB3D (NP_004274.1) was analyzed using Clustal 2.1 multiple sequence alignment (https://www.genome.jp/tools-bin/clustalw; accessed on 27 October 2021; Kyoto University Bioinformatics Center). The RNA expressional ratios of male germ cells (RAB3A, RABB, RAB3C, and RAB3D) were obtained from the original data of the single-cell RNA dataset of the Human Protein Atlas (https://www.proteinatlas.org/ENSG00000105649-RAB3A/single+cell+type; https://www.proteinatlas.org/ENSG00000169213-RAB3B/single+cell+type; https://www.proteinatlas.org/ENSG00000152932-RAB3C/single+cell+type; https://www.proteinatlas.org/ENSG00000105514-RAB3D/single+cell+type; accessed on 23 August 2021) [43]. RAB3B and RAB3C localization profiles on human testicular sections were collected from The Human Protein Atlas (https://www.proteinatlas.org/ENSG00000169213-RAB3B/tissue/testis#img; https://www.proteinatlas.org/ENSG00000152932-RAB3C/tissue/testis#img; accessed on 25 August 2021) [44].

### 2.2. Human Sperm Collection

The human semen collection protocol was approved by the Institutional Review Board of the Cathay General Hospital (No.: CGH-P102031). All clinical samples were obtained with informed consent from all participants. The participants were diagnosed using semen analysis according to the WHO laboratory manual for the examination of human semen (World Health Organization, 2010). The semen parameters met the standard (sperm concentration of ≥15 × 10^6^/mL, progressive motility of >32%, and normal morphology of >14%) as a control (normozoospermia; n = 116). The other participants had abnormal semen parameters (n = 254) [29]. More than 200 sperm were analyzed per participant. Morphological analysis was performed by sequencing the coding region of SEPT14, which identified the mutation sites in six participants with teratozoospermia (91.5% ± 2.88% abnormal sperm); morphological abnormalities were major in the sperm head (90% ± 4%) [29]. Sperm from participants with SEPT14 mutation (n = 6) and controls (n = 6) were subjected to immunofluorescence analysis.

### 2.3. Immunofluorescence Assay

Immunofluorescence analysis (IFA) was performed as described in our earlier studies [25,45,46]. Both human and murine male germ cells were treated with 0.1% Triton X-100 and washed twice with Tris-buffered saline (TBS). The washed slides were incubated with the primary antibody (TUBA: rabbit anti-α-tubulin antibody, Cat.Ab52866, Abcam, Cambridge, UK; anti-RAB3B/C antibody, Cat.15774-1-AP, Protintech, Rosemont, IL, USA). Further, the slides were washed four times with 0.02% TBS and incubated with an Alexa Fluor 488-labeled secondary antibody (Thermo Fisher Scientific, Waltham, MA, USA). Mitochondria were stained using MitoTracker (MitoTracker Red, Thermo Fisher Scientific, Waltham, MA, USA), and nuclei were counterstained with 4′,6-diamidino-2-phenylindole (DAPI). Fluorescent mounting medium (Dako, Fluka Analytical, München, Germany) was dispensed onto the slides, and glass coverslips were applied before evaluation by fluorescence microscopy. Slides were visualized using a fluorescence microscope (Olympus, Tokyo, Japan) under identical settings.

### 2.4. Collection of Mature Sperm and Isolation of Testicular Germ Cells

The animal studies were approved by the Institutional Animal Care and Use Committee of the Fu Jen Catholic University (A10577; 17 March 2017). Spermatogenic cells isolated from murine testicular tissues (n = 3) were separated based on the density of various germ cell types using a centrifugal system, as described in our previous studies [46]. After decapsulating, slicing, and digesting the testicular tissues with collagenase, suspension cells were filtered through 35-nm nylon filters (BD Falcon, Becton, Dickinson), then centrifugated using a Kubota 3330 centrifuge (Kubota Corp, Osaka, Japan). The separation fractions of spermatogonia, round spermatids, and secondary and primary spermatocytes were collected sequentially for 10 min at 750×, 420×, 190×, and 50× *g*, respectively. The purity of these specific cell types was examined under a light microscope after DAPI staining. Mature sperm were collected from the cauda epididymis of the adult mice. The epididymal cauda was sliced into small pieces in a petri dish containing 1× phosphate-buffered saline (PBS) to isolate epididymal spermatozoa. The collected solution was centrifuged for 10 min at 300× *g* to collect the spermatozoa pellet. The resuspended spermatozoa were air-dried on slides and subjected to IFA. 

### 2.5. Statistical Analysis

Spermatozoa were clustered according to the different types of morphological abnormalities. An unpaired *t*-test was used to compare the differences in the percentage of RAB3C localization and expressional levels among the different types of morphological abnormalities. A *p* value < 0.05 was considered significant. Prism statistical package was used for statistical analyses.

## 3. Results

### 3.1. RAB3C Is the Major Expressed Gene within the RAB3 Family during Human Spermatogenesis

An alignment assay was performed to evaluate the similarity between the amino acid sequences of human RAB3A, B, C, and D. The amino acids of the RAB3 family (RAB3A, RAB3B, RAB3C, and RAB3D) were highly conserved (Supplementary Figure S1). The resulting percentages between the RAB3 amino acid sequences ranged from 73.5–82.7%. To reveal the transcript expression of the RAB3 family in human testis, we collected the data from the RNA library and tissue arrays in the Human Protein Atlas. Among the transcriptomes of the human RAB3 family, RAB3C had the highest expression when compared with the other members (RAB3A, RAB3B, and RAB3D). In addition, the RAB3C transcripts are highly expressed at the late stages of human spermatogenesis, i.e., elongating and late elongating spermatids (Supplementary Figure S2A). In the human testicular sections, RAB3C proteins were located in spermatocytes (red arrows), early elongating (black arrows), and late spermatids (yellow arrows) (Supplementary Figure S2B–D). RAB3B protein was undetectable in the testicular sections (Supplementary Figure S3). Collectively, among the RAB3 family, RAB3C is mainly expressed and localized in post-meiotic human spermiogenesis.

### 3.2. RAB3C Protein Expression in the Mature Human Sperm

**Figure 1:**
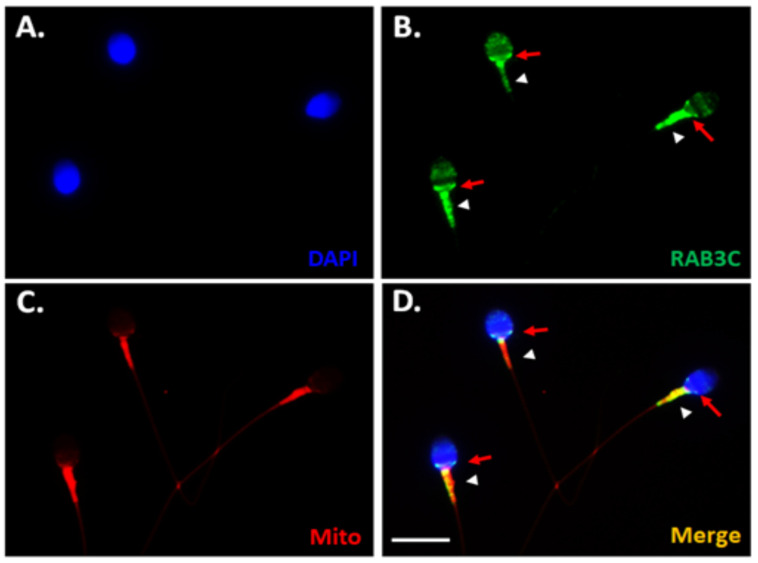
Localization of RAB3C in human spermatozoa. (**A**) Localization of sperm nuclei by DAPI staining. (**B**) Signals of RAB3C protein (green) localized at the postacrosomal region (red arrows) and midpiece (white arrowhead) in the human sperm. (**C**) MitoTracker (red) staining. (**D**) Merged immunofluorescence staining image (**A**–**C**). (Scale bar: 10 μm).

To determine the localization of RAB3C in mature human sperm, an IFA was performed. Co-staining with DAPI (nuclear dye, blue) and mitochondrial markers (Mitotracker, red) in Figure 1 demonstrates that RAB3C proteins were mainly localized at the postacrosomal regions of the sperm heads (Figure 1B, red arrows) and the midpiece region of the sperm tails. Thus, we revealed that RAB3C proteins were mainly localized in the postacrosomal and midpiece regions in the human spermatozoa.

### 3.3. Delocalized/Decreased RAB3C Signals in Sperm Harboring Mutated SEPT14

**Figure 2:**
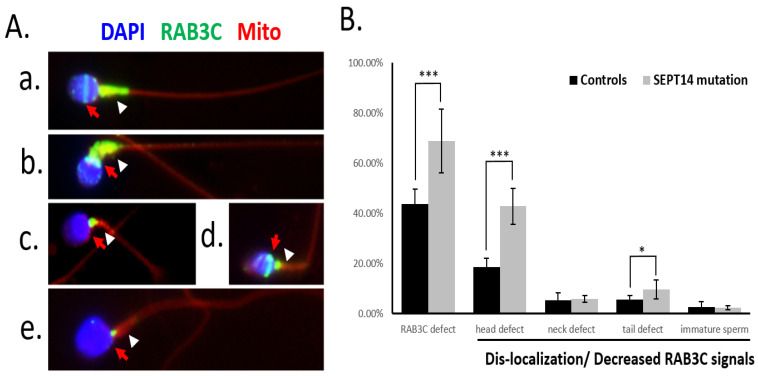
Delocalization/decreased RAB3C in the mutated *SEPT14*-harboring human sperm. (**A**) RAB3C protein signals are shown over the postacrosomal region (red arrow) and midpiece (white arrowhead) in normal human sperm (**a**) Spermatozoa from *SEPT14*-mutated sperm with neck (**b**,**c**), and tail defects (**d**) and head (**e**). DAPI, RAB3C, and mitochondria are labeled in blue, green, and red, respectively. (**B**) High percentages of delocalized/decreased RAB3C in the *SEPT14*-mutated sperm, compared with the healthy controls. The *Y*-axis indicates the frequency of delocalized or decreased RAB3C signals in spermatozoa (defined as the number of spermatozoa with delocalized or absent RAB3C signals/total number of spermatozoa). Black bars: controls (normozoospermic sperm); gray bars: SEPT14-mutated sperm. The abnormal spermatozoa morphologies were divided into four subgroups according to the following morphological criteria: head defects, neck and tail defects, and immaturity. (*** *p* < 0.001, * *p* < 0.05; unpaired *t*-test. Error bars indicate ± SD).

In our previous study, we discovered that the mutated SEPT14 resulted in teratozoospermia (n = 6), with >14% spermatozoa retaining their normal morphology [29]. We further identified SEPT14 interactors, a peptide of RAB3B/C [30]. In this study, Figure 1 and Supplementary Figure S2 suggest that RAB 3C is a major component of human sperm formation within the RAB3 family, and there is no RAB3B signal. To evaluate whether RAB3C is associated with the *SEPT14* mutation-induced sperm morphological abnormalities, we analyzed the localization of RAB3C in spermatozoa harboring these *SEPT14* mutations (Figure 2A). Figure 2 reveals that a higher percentage of the *SEPT14*-mutated sperm have delocalized/decreased RAB3C signals compared to the normozoospermic sperm without *SEPT14*-mutations (Figure 2B, left column). Among the diverted defects in sperm morphology, such as head, neck, and tail defects and immature sperm, we found that the head- and tail-defective sperm from the *SEPT14*-mutated cases exhibited higher percentages of delocalized/decreased RAB3C signals compared to the healthy controls (Figure 2B). Based on these results, we suggest that optimal RAB3C localization is associated with the molecular functions of SEPT14 during morphological development.

### 3.4. Dynamic Localization of RAB3C during Murine Spermiogenesis

**Figure 3:**
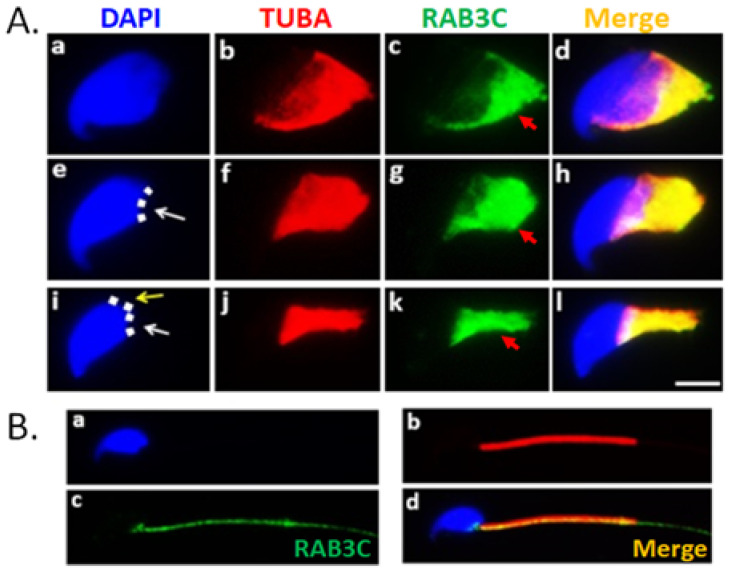
Dynamic localization of RAB3C during murine spermiogenesis. (**A**) Localization of RAB3C proteins in elongated spermatids during murine spermiogenesis. Sperm samples were stained with DAPI (nucleus, blue), and anti-TUBA (red) and anti-RAB3C (green) antibodies. The figures on the left indicate sperm head morphology at various stages. White and yellow arrows indicate ventral and dorsal angles, respectively. And, red arrows indicate RAB3C signals. The dotted lines indicate the sites of the angles. Spermatids at (**a**–**d**) step 7–9, (**e**–**h**) ventral angles at step 10, and (**i**–**l**) ventral and dorsal angles at step 11 during murine spermiogenesis. (**B**) RAB3C signals (green) are remarkably colocalized with MitoTracker (red) in the midpiece and postacrosomal regions of the mature sperm. (Magnification: 400×).

To evaluate the putative developmental role of RAB3C during sperm head and tail formation, isolated murine germ cells from testicular tissues were collected and subjected to immunostaining. RAB3C was first localized to the manchette structure, a transitional structure for the sperm head whose shape is formed by microtubules and actin filaments in early and late elongated spermatids during murine spermiogenesis (Figure 3A(a–h)). During the progression of sperm head formation, RAB3C became increasingly concentrated in the narrow manchette (Figure 3A(i–l)). Finally, RAB3C was located in the midpiece of the mature sperm (Figure 3B). These findings collectively suggest that RAB3C is involved in sperm head shaping and tail elongation during spermiogenesis.

## 4. Discussion

In this study, we found that RAB3C, a SEPT14 interactor, was localized in the human sperm head and midpiece regions, and the RAB3B protein was absent during human spermatogenesis. From a clinical perspective, high percentages of delocalized/decreased RAB3C were found in sperms harboring the mutated *SEPT14* than in the healthy controls. Analyzing the last morphological development of post-meiotic male germ cells suggested that the RAB3C signals were mainly involved in murine sperm head shaping and tail elongation. This study demonstrated the localization of RAB3C on male germ cells and the association between RAB3C and SEPT14 mutation-induced teratozoospermia.

### 4.1. RAB3 Family Expression on Human Spermatogenesis

Several RABs have been implicated in several biological stages of mammalian spermatogenesis [47,48]. For instance, RAB8B and RAB13 are localized in adherens junctions between Sertoli and male germ cells in mammalian testes, and they regulate trafficking events [49,50]. Furthermore, RAB10 is dynamically expressed in manchette structures and is involved in the formation of sperm heads in elongating spermatids [46]. Iida et al. observed that RAB3A was localized at the rat acrosomal membrane through immunostaining and immune-gold electron microscopy [42]. In human spermatozoa, RAB3A is also present in the acrosomal region of mature sperm [41]. However, the expression patterns of RAB3B, RAB3C, and RAB3D remain unknown. In this study, we found that the human RAB3B transcript level was low (Supplementary Figure S2A) in the different developmental stages of male germ cells, and the RAB3B protein was undetectable in human testicular sections (Supplementary Figure S3). The RNA transcripts of RAB3D were the lowest among the RAB3 family (Supplementary Figure S2A). Human RAB3C proteins are found in spermatocytes from the elongating to mature sperm stages, and RAB3C is specific to morphological transition stages (Supplementary Figure S2B–D). Isolated murine germ cells revealed that RAB3C is involved in sperm head and tail formation in elongating and elongated spermatids (Figure 3). Therefore, we present the first report of the RAB3C expressional patterns during human spermatogenesis that suggests its association with sperm head and tail formation.

### 4.2. Functional Roles of the RAB3 Family in Male Germ Cells

RAB3 family shares highly conserved sequences between RAB3A, RAB3B, RAB3C, and RAB3D. Several studies on RAB3 focus on the exocytosis of synaptic vesicles in neurons [35,36,37,39]. Overexpression of the Rab3 gene significantly decreases Ca^2+^-triggered exocytosis in cell models [39]. In male reproduction, a synthetic peptide of RAB3A inhibited acrosomal exocytosis triggered by the Ca^2+^ ionophore A23187 in a concentration-dependent manner [42]. Moreover, different regions of human RAB3A also exhibit the positive and negative effects on acrosomal reactions in streptolysin O-permeabilized human spermatozoa by Yunes et al. [41]. In knockout mice, all single and double knockouts of the Rab3 allele in male mice remained fertile [37,51]. This likely suggests the existence of highly complementary effects between the RAB3 members. The deletion of four RAB3 members led to the mice dying shortly after birth [51]. Moreover, homozygous RAB3B and RAB3C expression rescued the survival of quadruple KO mice. Furthermore, Rim is a critical effector of the RAB3 family that regulates synaptic vesicle fusion [38]. Deleting the Rim interactor, Rim-BP3, revealed severe defects in the sperm head [52]. This suggests that RAB3 proteins are involved in sperm morphology. In this study, we discovered that RAB3C was mainly localized in the postacrosomal region of the sperm head and the midpiece of mature human spermatozoa (Figure 1). In addition, the dynamic localization of RAB3C is in the manchette structure and tail during murine sperm head shaping and axonemal structure formation in the tail (Figure 3). This is, to the best of our knowledge, the first report of the possible RAB3C roles during sperm formation. 

### 4.3. Clinical Views of RAB3C Protein and SEPT14 Mutated Caused Teratozoospermia

From a clinical perspective, semen quality, which is adjudged by parameters including sperm concentration, motility, and morphology, is a major factor associated with ART outcomes [8,9]. Specifically, poor sperm morphology scores affect the success rate of pregnancy more than sperm concentration and motility. Furthermore, abnormalities in morphology, including sperm head defects, usually accompanied by DNA/nuclear damage, are mainly associated with the negative effects on ART results [8,9,10,11,12]. Several genetic mutations, including *PRM1*, *AURKC*, *SPATA16*, *PICK1*, *DPY19L2*, *SEPT12*, and *SEPT14*, result in male infertility and are caused by sperm DNA damage in a clinical context [4,53,54]. In our previous studies, we identified mutations in the *SEPT12* and *SEPT14* genes by sequencing their coding regions and showed that they caused severe sperm head deformation and DNA damage in male infertility cases [26,29]. We also found that SEPT12 and SEPT14 form filamentous structures and assist in sperm head shaping and tail elongation during human spermiogenesis [25,26,29,30,55]. *SEPT12* or *SEPT14* mutations disrupt filamentous structures, causing morphological defects in sperm and DNA damage [25,30]. In this study, we confirmed that RAB3C is localized in the human sperm head and midpiece of the sperm tail. Furthermore, we found that spermatozoa with mutated *SEPT14* exhibited delocalized/decreased RAB3C signals compared to the normozoospermic sperm without *SEPT14*-mutations (Figure 2). Moreover, the limitation that the localization of the RAB3C expression was not evaluated between the *SEPT14*-mutated and the *SEPT14*-normal spermatozoa with abnormal morphology in this study. We propose that RAB3C, a SEPT14 interactor, is involved in the molecular function of SEPT14 during sperm formation. Moreover, RAB3C may be a good biomarker for morphological defects in sperm.

## 5. Conclusions

In conclusion, the aforementioned findings suggest a new link between the RAB3C protein and the *SEPT14* mutations, which causes morphological sperm head and tail defects.

## Data Availability

Not applicable.

## Supplementary Materials

The following supporting information can be downloaded at: https://www.mdpi.com/article/10.3390/medicina58101408/s1, Figure S1. Alignment of Human RAB3A, RAB3B, RAB3C, and RAB3D amino acid sequences. Figure S2. Expression profiles and localization of RAB3C in human spermatogenesis. Figure S3. Lack of RAB3B signaling at the differential stages of human post-meiotic male germ cells from the Human Protein Atlas.
