# Supplementary material for: Localization Patterns of RAB3C Are Associated with Murine and Human Sperm Formation
Source: Medicina (Kaunas). 2022 Oct 7;58(10):1408. doi: 10.3390/medicina58101408 (PMC9606999; doi:10.3390/medicina58101408)
Supplement: Supplementary file 1 [file medicina-58-01408-s001.zip › medicina-1818612-supplementary.pdf]

```
RAB3A      --------MASATDSRYGQKESSDQNFDYMFKILIIGNSSVGKTSFLFRYADDSFTPAFV
RAB3B      --------MASVTDGKTGVKDASDQNFDYMFKLLIIGNSSVGKTSFLFRYADDTFTPAFV
RAB3C      MRHEAPMQMASAQDARYGQKDSSDQNFDYMFKLLIIGNSSVGKTSFLFRYADDSFTSAFV
RAB3D      --------MASAGDTQAGPRDAADQNFDYMFKLLLIGNSSVGKTSFLFRYADDSFTPAFV
                   ***. * : * ::::*********:*:******************:**.***

RAB3A      STVGIDFKVKTIYRNDKRIKLQIWDTAGQERYRTITTAYYRGAMGFILMYDITNEESFNA
RAB3B      STVGIDFKVKTVYRHEKRVKLQIWDTAGQERYRTITTAYYRGAMGFILMYDITNEESFNA
RAB3C      STVGIDFKVKTVFKNEKRIKLQIWDTAGQERYRTITTAYYRGAMGFILMYDITNEESFNA
RAB3D      STVGIDFKVKTVYRHDKRIKLQIWDTAGQERYRTITTAYYRGAMGFLLMYDIANQESFAA
           ***********:::::**:***************************:*****:*:*** *

RAB3A      VQDWSTQIKTYSWDNAQVLLVGNKCDMEDERVVSSERGRQLADHLGFEFFEASAKDNINV
RAB3B      VQDWATQIKTYSWDNAQVILVGNKCDMEEERVVPTEKGQLLAEQLGFDFFEASAKENISV
RAB3C      VQDWSTQIKTYSWDNAQVILVGNKCDMEDERVISTERGQHLGEQLGFEFFETSAKDNINV
RAB3D      VQDWATQIKTYSWDNAQVILVGNKCDLEDERVVPAEDGRRLADDLGFEFFEASAKENINV
           ****:*************:*******:*:***:.:* *: *.:.***:***:***:**.*

RAB3A      KQTFERLVDVICEKMSESLDTADPAVTGAKQGPQLSDQQVPPHQDCAC
RAB3B      RQAFERLVDAICDKMSDSLDT-DPSMLGSSKNTRLSDTPPLLQQNCSC
RAB3C      KQTFERLVDIICDKMSESLET-DPAITAAKQNTRLKETPPPPQPNCAC
RAB3D      KQVFERLVDVICEKMNESLEP-SSSSGSNGKGPAVGDAPAPQPSSCSC
           :*.****** **:**.:**:. ..:  .  :.. : :       .*:*
```

Figure S1. Alignment of Human RAB3A, RAB3B, RAB3C, and RAB3D amino acid sequences. Alignment of the RAB3 family amino acids is highly conserved between human RAB3A, RAB3B, RAB3C, and RAB3D. Identical amino acids are highlighted with an asterisk (*). Highly conserved amino acids are marked with dots (:) and (.), suggesting that one or two amino acids are unidentical, respectively.

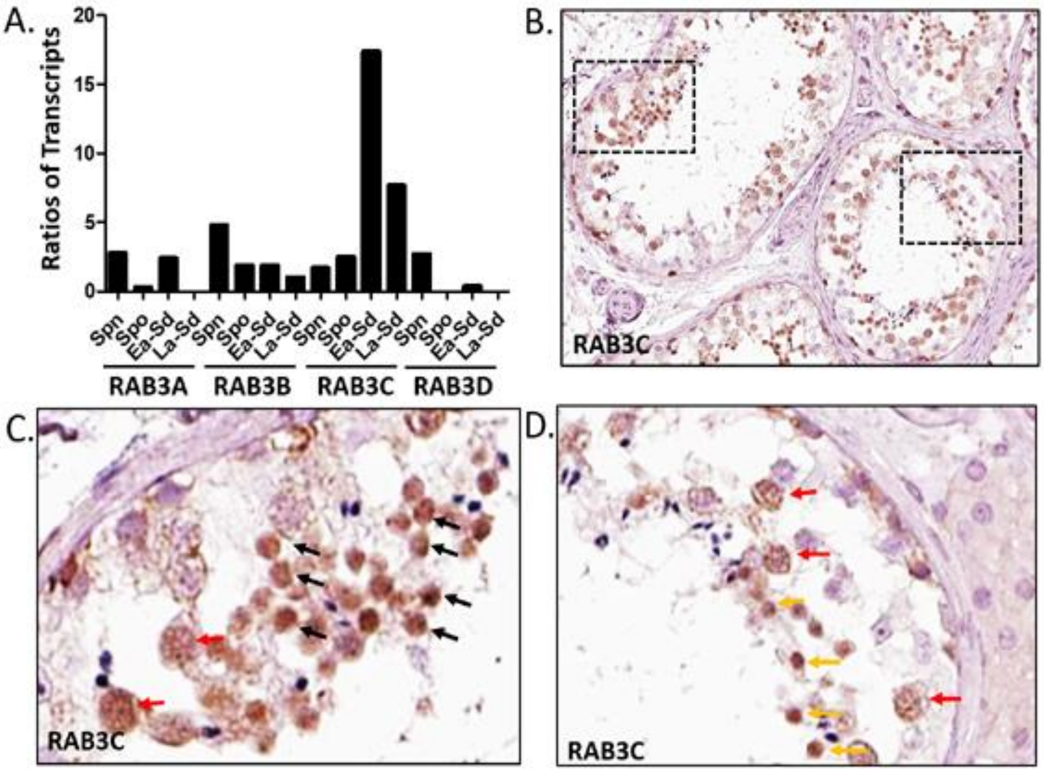

Figure S2. Expression profiles and localization of RAB3C in human spermatogenesis. (A) The transcripts of RAB3A, RAB3B, RAB3C, and RAB3D are shown at different stages of human post-meiotic male germ cells from The Human Protein Atlas. (B–D) Immuno-histochemical results of RAB3C on human testicular sections collected from The Human Protein Atlas. (C, D) represent the dotted regions enlarged from Figure 2B. Red, black, and yellow arrows indicate spermatocytes, early and late spermatids, respectively.

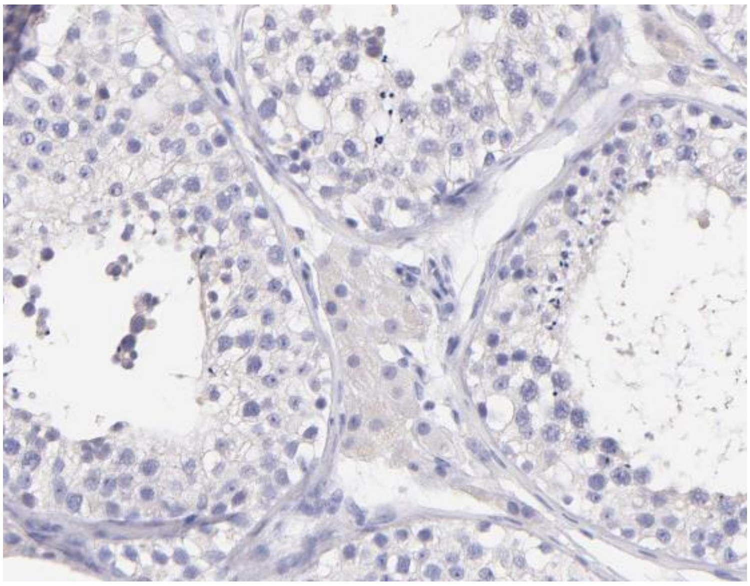

Figure S3. Lack of RAB3B signaling at the differential stages of human post-meiotic male germ cells from the Human Protein Atlas. The figure is depicted by staining with an anti-RAB3B antibody.
